# Optimization of Fermentation Conditions for *Bacillus amyloliquefaciens* JL54 and Preparation of Powder Through Spray Drying

**DOI:** 10.3390/plants14081263

**Published:** 2025-04-21

**Authors:** Leilei Zhao, Yanru Wang, Min Pan, Weiliang Kong, Haifeng Wang, Jiajin Tan

**Affiliations:** 1 Collaborative Innovation Center of Modern Forestry in South China, College of Forestry and Grassland, Nanjing Forestry University, Nanjing 210037, China; leileizhaoo@163.com (L.Z.); wangyanru@njfu.edu.cn (Y.W.); pam@njfu.edu.cn (M.P.); k3170100077@njfu.edu.cn (W.K.); 2 Forestry Bureau of Dunhua City, Dunhua 133703, China; dhjianyi@163.com

**Keywords:** *Bacillus amyloliquefaciens*, larch blight, response surface methodology, fermentation optimization

## Abstract

Larch dieback disease, caused by *Neofusicoccum laricinum*, severely affects forest health and productivity. To effectively curb the occurrence of this disease, a powder formulation of *Bacillus amyloliquefaciens* JL54 was developed through spray drying. The research commenced with the optimization of fermentation medium and culture conditions through statistical design, aiming to maximize both bacterial viability and antagonistic activity. The optimal medium included 12 g/L yeast extract, 11.8 g/L yeast powder, and 7.2 g/L magnesium sulfate. Optimal culture conditions included 30% loading volume, 1% inoculum, 37 °C incubation temperature, 31.8 h shaking time, and initial pH 6.4. Under these conditions, the viable count of strain JL54 reached 4.45 × 10^9^ cfu/mL, a 296.67-fold increase compared with the unoptimized system. To evaluate its practical applicability, field trials were conducted, showing a 54% control efficiency against larch dieback disease, significantly suppressing disease progression. Subsequently, the spray drying process was optimized with a 1:10 protective agent-to-water ratio, 100 °C inlet temperature, and 630 mL/h feed flow rate, achieving a 78.41% powder recovery rate. Collectively, this study demonstrates the potential of *B. amyloliquefaciens* JL54 as an effective biocontrol agent for managing larch dieback and supports its application in spray-dried formulations for forest disease control.

## 1. Introduction

*Larix* spp. exhibit notable adaptability to diverse environments, characterized by high cold resistance, preference for sunlight, and rapid growth rates. *Larix* spp. wood possess a strong substance, a detailed structure, low causticity, and high economic worth. Because of these features, *Larix* spp. are usually chosen as the preferred tree species for fast-growing high-yield artificial timber forests.

Larch plantations are seriously threatened by larch shoot blight, which is regarded as one of the most serious forest diseases in the world [1]. In 1938, it was first discovered in Hokkaido, Japan [2,3]. The pathogen was identified as *Physalospora laricina* Sawada in 1950 [4]. Subsequently, in 1961, it was recombined by Yamamoto and Kazuo to become *Guignardia laricina* (Sawada) W. Yamam. & Kaz. Itô [2,5]. Since the 1970s, many Chinese researchers have studied the disease from different perspectives, and they all believe that Chinese larch dieback is the same as Japanese larch dieback. As a result, the pathogen’s scientific name also follows Japanese usage. In 2021, Hattori and Nakashima suggested reclassifying the strain as *Neofusicoccum laricinum* (Sawada) Y. Hattori & C. Nakash, indicating a significant advancement in this pathogen’s taxonomy [1]. 

This disease is spread throughout parts of China, Japan, South Korea, North Korea, Russia, and other countries. A significant fungal disease that is common in larch plantations in northern China is larch shoot blight, which is distinguished by its rapid propagation and extreme destructiveness. Diseased buds, needles, branches, roots, and stems may droop and bend as a result of the infection, eventually shriveling. The plant may wilt and, in extreme circumstances, perish as a result of the diseased sections spreading to the branches [6]. Due to its considerable impact, China recognized it as an official quarantine object in 2013 [7] and a major invasive alien species for management in 2023 [8]. Additionally, the European and Mediterranean Plant Protection Organization has classified the pathogen as a quarantine pest [9].

The incidence of larch shoot blight has significantly increased in recent years, posing a major risk to larch. At the moment, chemical control and the implementation of appropriate procedures for managing forests are the main methods of both avoiding and treating the disease. Nevertheless, the disease cannot be prevented using these treatments. However, biological control is being utilized more often in an effort to reduce the pollution that chemical pesticides produce. Plant-beneficial or harmless organisms are used to control or inhibit the spread and survival of pathogens [10]. As a result, biological control is a crucial component of comprehensive management and one of the key strategies for illness prevention and control. The biological control of pine shoot blight by using endophytic bacteria has the advantages of not causing pollution to the environment and resistance to fungicides, it also does not threaten the stability of forest ecosystems. As a result, this control strategy offers several clear benefits and has been crucial in the fight against numerous plant diseases [11].

*Bacillus* spp. are found in many different species and organisms, including plant tissues, leaves, and roots [12]. *Bacillus* is Gram-positive bacterium that is highly adaptable to extreme environmental conditions (such as high temperature and osmosis) [13]. *Bacillus* can produce a range of biologically active bacteriostatic substances, including lipopeptide antibiotics and antimicrobial peptides [14,15,16]. These substances can be used as biopesticides to prevent postharvest diseases in fruits and vegetables by producing bacteriostatic substances [17] and to inhibit the growth of pathogenic bacteria through antagonism, competitive action, and induced resistance [18,19].

Studies have shown that *Bacillus amyloliquefaciens* has the characteristics of non-pathogenicity, strong resistance, a rich variety of inhibitory secondary metabolites, a wide antimicrobial spectrum, and the ability to improve the growth performance and disease resistance of crops, which makes it one of the ideal microorganisms for the biocontrol of plant diseases [20]. *B. amyloliquefaciens*’s capacity to control plant diseases is linked to the production of antimicrobial proteins, lipopeptides, and polyketides. Among these bacteriostatic active substances, lipopeptides have attracted much attention because of their high efficiency, stability, and low toxicity. Surfactin, iturin, and fengycin are the primary lipopeptide molecules with strong antibacterial properties [21].

In recent years, there have been increasing research reports on the prevention and control of plant diseases by *B. amyloliquefaciens*, but there is a lack of reports on its commercialization and industrial exploitation and utilization. The results of Wang Rui et al. [22] showed that *Bacillus velezensis* wr8 was able to antagonize *Penicillium* sp. to control postharvest citrus rot. Isolated from the soil, *B. velezensis* Jnb16 can effectively inhibit *Magnaporthe oryzae* (the rice blast fungus) and has a good growth-promoting effect on rice [23]. It can also control root rot in strawberries [24], gray mold in cucumbers [25], and apple tree rot [26]. In addition, *B. amyloliquefaciens* plays a role in biological and chemical degradation [27]. Therefore, the application of *B. amyloliquefaciens* bacterial agents instead of chemical pesticides in agroforestry production can not only inhibit the occurrence of certain plant diseases and maintain the stability of the ecosystem but also reduce the adverse effects of chemical pesticides on human health and the environment, which has a broad prospect for development.

In practical production applications, solid bacterial powder is in a typical dose form. Prepared from the original bacterial liquid, carrier, surfactant, and other additives, it is low-cost, needs to be mixed and crushed into a fine dry agent, and overcomes the disadvantages of water-based formulations in terms of storage and transportation. It also has the advantages of solid formulations, such as stable efficacy, ease of use, and so on [28]. Wettable powders of *B. amyloliquefaciens* have a wide range of applications. The B15 wettable powder, which was made by Jin et al., provided effective protection against grapevine diseases and had potential value in the field of fruit and vegetable preservative applications [29]. *B. amyloliquefaciens* X17 wettable powder was produced by Qian et al. and proved to be substantially more effective than the initial fermented powder at preventing *Botrytis cinerea* in kiwifruits [30]. CC09 wettable powder has been shown to be more effective at controlling wheat powdery mildew [31]. With its high drying rate and strong product dispersion performance, spray drying technology is a good approach to making powders and has been used extensively [32,33]. It is an effective drying method to process liquid into powder products that covers practically all processing and production industries.

A strain of *B. amyloliquefaciens* JL54 was isolated from larch with an antagonistic effect against the pathogen of larch dieback disease. The strain was endophytic and was able to colonize and multiply well in larch [34]. The objectives of this study were to establish a cost-effective fermentation system maximizing the biomass and biocontrol potential of *B. amyloliquefaciens* JL54, and to develop a powder formulation with enhanced stability and field applicability through spray drying. It was hypothesized that optimizing both fermentation and spray drying processes would synergistically enhance bacterial viability and powder recovery efficiency, thereby increasing its efficacy as a sustainable biocontrol agent against larch dieback disease. To further understand the optimal fermentation conditions of *B. amyloliquefaciens* JL54, this study systematically optimized the medium components and fermentation conditions of strain JL54 using the response surface design method [35]. Then, a JL54 bacterial suspension was used as the material to obtain bacterial powder by the spray drying method, and the process conditions of spray drying technology were optimized by designing one-way and orthogonal tests. Using the number of viable bacteria and the powder collection rate as indexes to determine the optimal spray drying process parameters lays the foundation for the industrial production and popularization of strain JL54 in the future.

## 2. Results

### 2.1. Analysis of Optimization Results of Strain Fermentation Medium Components

#### 2.1.1. Analysis of Carbon Source Optimization Results

**Figure 1:**
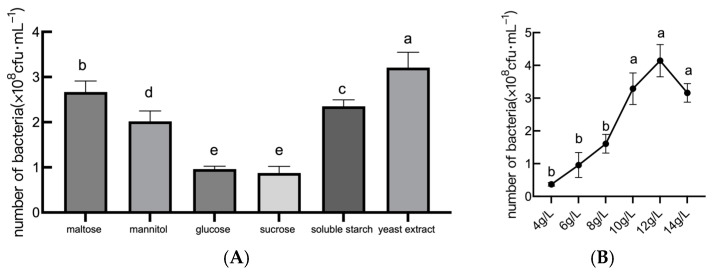
Effects of different carbon sources on the growth of *B. amyloliquefaciens*. Note: (**A**) the number of viable *B. amyloliquefaciens* JL54 bacteria with different carbon source culture media; (**B**) the number of viable bacteria in the fermentation filtrate of *B. amyloliquefaciens* JL54 with different yeast extract concentrations. Data are presented as means of three replicates ± SD, and error bars represent SD for three replicates. Means with different letters have significant differences (*p* < 0.05; Duncan test).

The carbon source selection significantly influenced the growth of *B. amyloliquefaciens* JL54. In Figure 1A, the number of viable *B. amyloliquefaciens* JL54 bacteria present in the various carbon sources (yeast extract, maltose, soluble starch, mannitol, glucose, and sucrose) is shown. Yeast extract supported the maximum bacterial viability (3.31 × 10^8^ cfu/mL), followed by maltose and soluble starch. In contrast, sucrose yielded the lowest viable count. It is evident from Figure 1B that JL54 grew differently at different yeast extract concentrations. A dose-dependent response was found when the concentration of yeast extract was further optimized and the maximum viability (4.1 × 10^8^ cfu/mL) was reached at 12 g/L. Notably, concentrations exceeding 12 g/L led to a slight decline, so it was appropriate to use this concentration as the carbon source concentration for *B. amyloliquefaciens* JL54’s fermentation test.

#### 2.1.2. Analysis of Nitrogen Source Optimization Results

**Figure 2:**
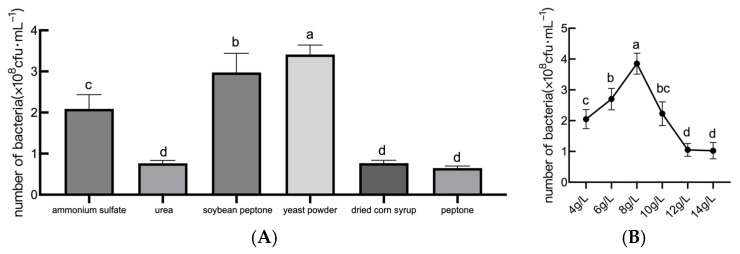
Effects of different nitrogen sources on the growth of *B. amyloliquefaciens*. Note: (**A**) the number of viable JL54 bacteria with different nitrogen source culture media; (**B**) the number of viable bacteria in the fermentation filtrate of *B. amyloliquefaciens* JL54 with different yeast powder concentrations. Data are presented as means of three replicates ± SD, and error bars represent SD for three replicates. Means with different letters have significant differences (*p* < 0.05; Duncan test).

Nitrogen sources exhibited distinct impacts on bacterial growth, and peptone, yeast powder, urea, soybean peptone, ammonium sulfate, and dried corn syrup were selected as the medium nitrogen sources, respectively. Based on the analysis of Figure 2A, yeast powder outperformed other nitrogen sources, yielding 4.5 × 10^8^ cfu/mL, followed by soybean peptone and ammonium sulfate. As can be seen in Figure 2B, strain JL54 grew differently with different amounts of yeast powder. Viability peaked at 8 g/L yeast powder (4.62 × 10^8^ cfu/mL), but concentrations over 8 g/L decreased growth by 12–18%, suggesting nutrient saturation. Thus, for the *B. amyloliquefaciens* JL54 fermentation test that followed, yeast powder at a concentration of 8 g/L was used as the nitrogen supply concentration.

#### 2.1.3. Analysis of Inorganic Salt Optimization Results

**Figure 3:**
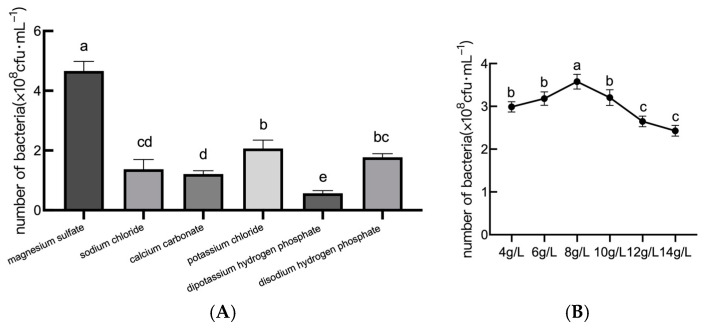
Effects of different inorganic salt sources on the growth of *B. amyloliquefaciens*. Note: (**A**) the number of viable *B. amyloliquefaciens* JL54 bacteria with different inorganic salt source culture media; (**B**) the number of viable bacteria in the fermentation filtrate of *B. amyloliquefaciens* JL54 with different magnesium sulfate concentrations. Data are presented as means of three replicates ± SD, and error bars represent SD for three replicates. Means with different letters have significant differences (*p* < 0.05; Duncan test).

As shown in Figure 3A, among six tested inorganic salt sources (magnesium sulfate, sodium chloride, calcium carbonate, potassium chloride, dipotassium hydrogen phosphate, and disodium hydrogen phosphate), magnesium sulfate emerged as the most effective inorganic salt, supporting a viable count of 4.94 × 10^8^ cfu/mL. Other salts, such as sodium chloride and calcium carbonate, showed inferior performance, so it is appropriate to choose magnesium sulfate as the best inorganic salt for the growth of JL54 bacterial organisms. From Figure 3B, it can be seen that optimization of the magnesium sulfate concentration revealed a limited optimal range (6 g/L maximized viability (5.2 × 10^8^ cfu/mL)), while deviations by ±2 g/L reduced counts by 15–22%. So, it is appropriate to select the magnesium sulfate concentration of 6 g/L as the concentration of inorganic salts for the subsequent fermentation test of *B. amyloliquefaciens* JL54.

### 2.2. Analysis of Optimization Results of Strain Fermentation Conditions

Critical fermentation parameters were systematically evaluated.

**Figure 4:**
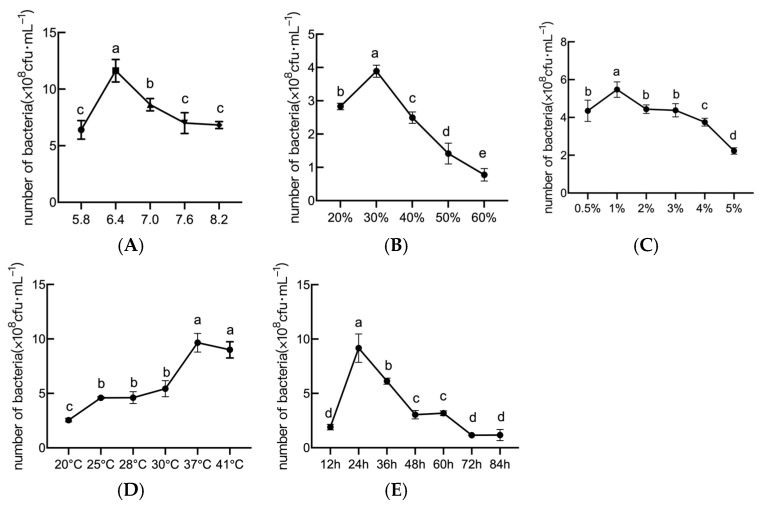
Effect of different culture conditions on the growth of *B. amyloliquefaciens* JL54. (**A**) The number of viable *B. amyloliquefaciens* JL54 bacteria at different initial pHs; (**B**) the number of viable *B. amyloliquefaciens* JL54 bacteria at different loading volumes; (**C**) the number of viable *B. amyloliquefaciens* JL54 bacteria at different inoculum volumes; (**D**) the number of viable *B. amyloliquefaciens* JL54 bacteria at different temperatures; (**E**) the number of viable *B. amyloliquefaciens* JL54 bacteria at different shaking times. Data are presented as means of three replicates ± SD, and error bars represent SD for three replicates. Means with different letters have significant differences (*p* < 0.05; Duncan test).

The results of initial pH screening (Figure 4A) indicated that the initial pH exhibited a pronounced influence on *B. amyloliquefaciens* JL54 viability, and growth peaked at pH 6.4 (1.21 × 10^8^ cfu/mL). However, deviations beyond this optimal range (6.1–7.0) resulted in reduced growth. Consequently, a pH of 6.4 was selected for the subsequent culture of strain JL54 growth and fermentation.

The results of the loading volume screening (Figure 4B) indicated that a 30% loading volume maximized bacterial density (3.89 × 10^8^ cfu/mL). Therefore, the loading volume of 30% was selected for the subsequent growth and fermentation of strain JL54.

The results of the inoculum screening (Figure 4C) indicated that a 1% inoculum yielded the highest viability (5.48 × 10^8^ cfu/mL), whereas larger inoculum (≥3%) reduced growth by 25–30%. Therefore, the inoculum volume for the subsequent culture of strain JL54 growth fermentation should be 1%.

The results of the temperature screening (Figure 4D) indicated that the number of viable bacteria exhibited a significant change at different temperatures. While JL54 tolerated a broad range (20–41 °C), optimal biomass accumulation (9.6 × 10^8^ cfu/mL) occurred at 37 °C. Therefore, this temperature was selected for the subsequent culture of strain JL54 growth and fermentation.

The results of the shaking time screening (Figure 4E) indicated that continuous shaking for longer than 24 h (up to 28 h) prolonged the exponential phase, but longer periods (>32 h) led to a decline. Therefore, the shaking time for the growth and fermentation of strain JL54 was selected to be 24 h for the subsequent culture.

### 2.3. Response Surface Optimization of Fermentation Culture of B. amyloliquefaciens JL54

#### 2.3.1. Plackett–Burman Test

The Plackett–Burman test was designed using Design Expert 10 software, and the results and analysis are presented in Table 1. The model exhibited an R^2^ of 98.56% and an R^2^_adj_ of 94.72%, with a *p*-value of 0.0110, which was less than 0.05, indicating that the model was more reliable. As illustrated in Table 1, an inverse relationship was observed between the levels of variables such as magnesium sulfate concentration and shaking bottle temperature, whereas a positive correlation was evident between the levels of variables such as yeast powder concentration, yeast extract concentration, shaking bottle time, initial pH, inoculum volume, and loading volume.

The order of significance of the effects on the growth of *B. amyloliquefaciens* JL54 was B > F > C > H > A > G > D > E. This indicates that the order of importance was yeast powder concentration > shaking time > magnesium sulfate concentration > liquid loading > yeast extract concentration > shaking temperature > initial pH > inoculum amount. The three factors with the most significant effects, namely yeast powder concentration (B), shaking time (F), and magnesium sulfate concentration (C), had contributions of 28.77%, 25.10%, and 19.43%, respectively, so the three factors were subjected to the next steepest climb test.

#### 2.3.2. Steepest Climb Test

As shown in Table 2, building on the PB results, the steepest climb test refined the optimal ranges for the three key factors. Yeast powder concentration and shaking time had positive effects and should be gradually increased in the steepest climb test (yeast powder 8→13 g/L and shaking time 24→34 h), whereas magnesium sulfate concentration had a negative effect and should be gradually decreased (magnesium sulfate 3→8 g/L). The results of the test indicate that treatment 5 (12 g/L yeast powder, 32 h shaking, 7 g/L magnesium sulfate) achieved the highest count (4.05 × 10^9^ cfu/mL). Therefore, the conditions of treatment 5 should be selected as the center point of the factor level of the response surface test.

#### 2.3.3. Box–Behnken Design (BBD) Analysis

The yeast powder concentration, shaking time, and magnesium sulfate concentration in the steepest climb test were designated as A, B, and C, respectively. The results and analysis of the BBD test are presented in Table 3. The Box–Behnken model demonstrated an R^2^ value of 93.76%, an R^2^_adj_ of 85.75%, a *p*-value of 0.0019, and a misfit term with a *p*-value greater than 0.05. These results indicate that the model was reliable and could be used for further analysis and prediction.

The model was used to analyze and predict the response values and it confirmed quadratic effects of yeast powder (A^2^, *p* < 0.01) and magnesium sulfate (C^2^, *p* < 0.01) on JL54 growth, while A, B, and C^2^ had a non-significant effect (*p* < 0.10). The test results were analyzed using Design Expert 10 software, which generated a quadratic regression equation for the response value and test factor. This equation was as follows:

$$\mathrm{quantity}\;\mathrm{of}\;\mathrm{viable}\;\mathrm{bacteria}=+3.38-0.24A-0.050B+0.36C+0.15AB-0.12AC-0.050BC-0.70A^{2}-0.43B^{2}-1.00C^{2}$$

**Figure 5:**
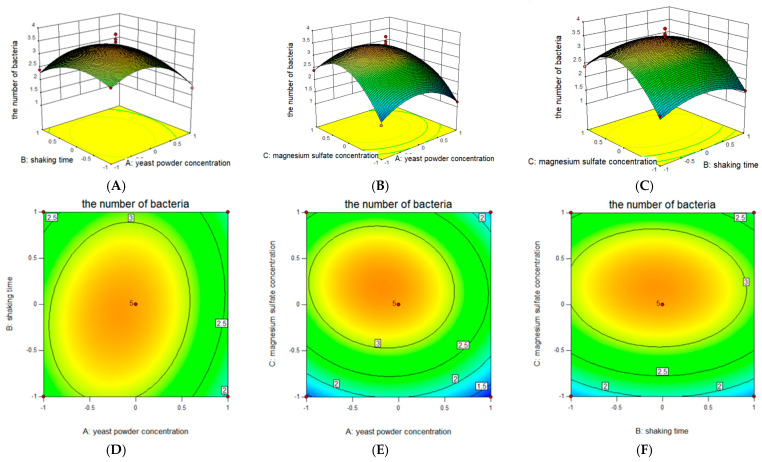
Response surface (**A**) and contour plots (**D**) show the effects of yeast powder concentration, shaking time, and their mutual interaction on the number of bacteria; response surface (**B**) and contour plots (**E**) show the effects of yeast powder concentration, magnesium sulfate concentration, and their mutual interaction on the number of bacteria; response surface (**C**) and contour plots (**F**) show the effects of shaking time, magnesium sulfate concentration, and their mutual interaction on the number of bacteria.

Response surface and contour plots were generated according to the regression equations, and the results are presented in Figure 5. The contour lines of yeast powder concentration and magnesium sulfate concentration (Figure 5B,E) were nearly circular, indicating that the interaction of yeast concentration and magnesium sulfate concentration did not have a significant effect on the amount of viable bacteria. The contour lines of yeast powder concentration and shaking time (Figure 5A,D) were approximately elliptical, indicating that the interaction of yeast concentration and shaking time had a significant effect on the amount of viable bacteria. The contour lines of magnesium sulfate concentration and shaking time (Figure 5C,F) were approximately elliptical, indicating that the interaction of magnesium sulfate concentration and shaking time had a significant effect on the amount of viable bacteria. The derived regression equation predicted optimal conditions: 12 g/L yeast extract, 11.8 g/L yeast powder, and 7.2 g/L magnesium sulfate, 30% loading, 1% inoculum, 37 °C incubation temperature, 31.8 h shaking time, and an initial pH of 6.4. Experimental validation confirmed a viable count of 4.45 × 10^9^ cfu/mL, aligning closely with the model’s prediction.

### 2.4. Comparison of Viable Bacteria Numbers in Fermentation Broth Before and After Fermentation Optimization

**Figure 6:**
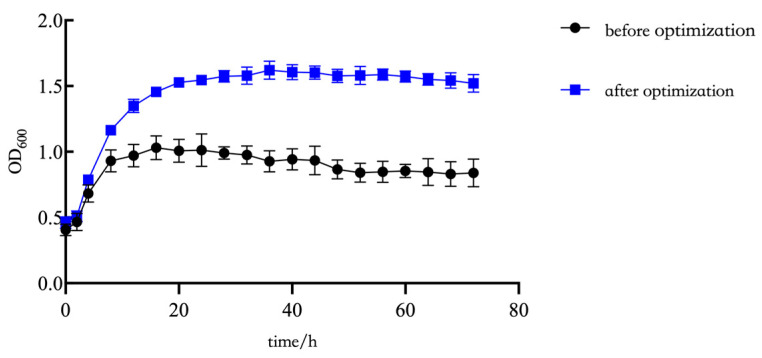
The OD600 of 72 h bacterial growth before and after optimization.

As shown in Figure 6, based on the culture parameters used before and after optimization for JL54, the growth of bacteria in the fermentation broth was observed to be slow for the first two hours; after that, the strain entered the exponential period, which was extended by eight hours compared with the exponential period prior to optimization. Finally, the strain entered the stabilization period after 28 h.

After a 24 h shake flask fermentation of the strain JL54 seed solution using optimized medium and shake flask conditions, the results of the gradient dilution plate coating revealed that the optimized fermentation broth’s average viable bacterial count was 4.45 × 10^9^ cfu/mL, approximately 296.67 times higher than the fermentation broth from the pre-optimization.

### 2.5. Field Experiments

**Figure 7:**
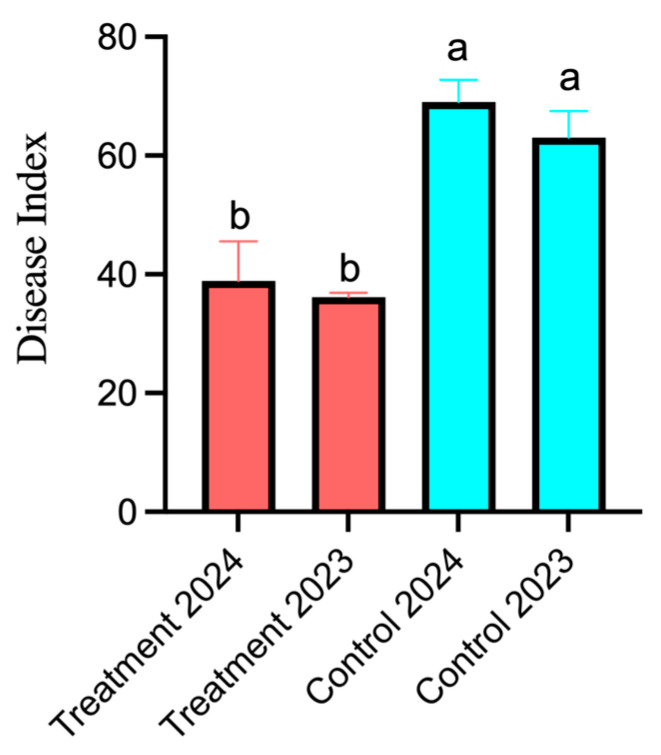
Comparison of 2023 and 2024 disease index mean values. Data are presented as means of three replicates ± SD, and error bars represent SD for three replicates. Means with different letters have significant differences (*p* < 0.05; Duncan test).

As Table 4 and Figure 7 show, the two studies of the disease index following the 2023 fungicide-free and 2024 fungicide-applied groups discovered that the treatment group’s disease index growth value decreased in comparison with the control group’s growth value. The disease index in the treatment group increased from 36.17 (2023) to 38.87 (2024), whereas the control group worsened from 63.03 to 68.98. This divergence (Δ = 2.7 vs. Δ = 5.95) highlighted that the *B. amyloliquefaciens* JL54 bacterial suspension had better control of the larch dieback disease developmental process, with a 54% field control effect. These results confirm the potential of *B. amyloliquefaciens* JL54 as a field-applicable biocontrol agent.

### 2.6. Results of Spray Drying Single Factor Test

#### 2.6.1. The Effect of Protective Agent Type

**Figure 8:**
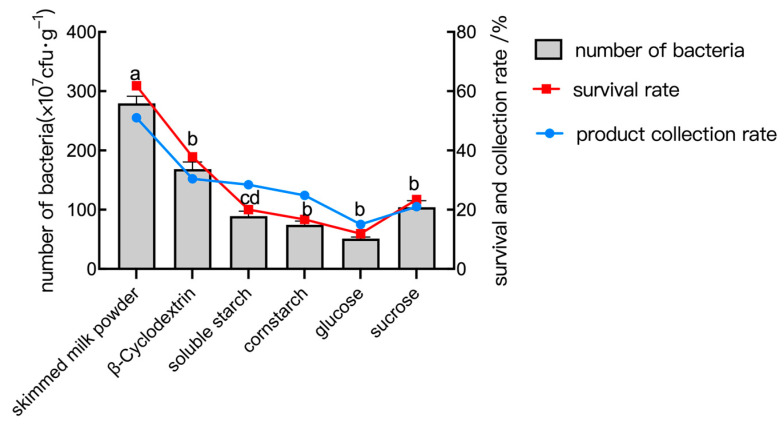
The number of bacteria, survival rate, and collection rate of the powder under different types of protective agent conditions. Data are presented as means of three replicates ± SD, and error bars represent SD for three replicates. Means with different letters have significant differences (*p* < 0.05; Duncan test).

Skimmed milk powder and other materials can provide a protective layer on the bacterium’s surface during the spray drying process, lessening the harm that the high temperature causes to the bacterium and increasing the product’s viability. According to the test, when glucose, sucrose, cornstarch, and soluble starch were used as protective agents, less powder was collected. In this case, glucose was more serious than sucrose, which would stick to the wall, while cornstarch and soluble starch would precipitate and obstruct the instrument’s feed tube. As shown in Figure 8, skimmed milk powder outperformed other agents, achieving a bacteria amount of 2.80 × 10^9^ cfu/mL, a survival rate of 78.2%, and a product collection rate of 65.4%.

#### 2.6.2. The Effect of the Material/Liquid Ratio

**Figure 9:**
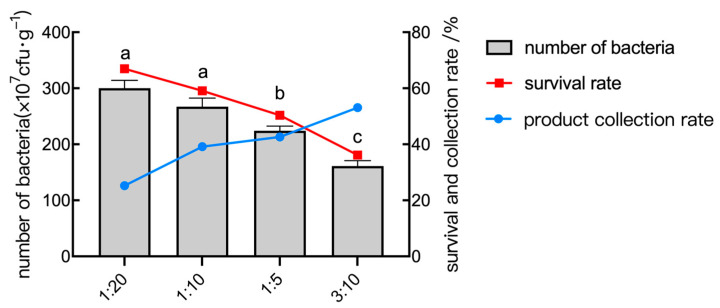
The number of bacteria, survival rate, and collection rate of the powder under different material/liquid ratio conditions. Data are presented as means of three replicates ± SD, and error bars represent SD for three replicates. Means with different letters have significant differences (*p* < 0.05; Duncan test).

As the amount of protective agent increased during the spray drying process, the number of live bacteria decreased, while the powder collection rate increased after spray drying. It can be seen from Figure 9 that, when the protective agent exceeded a certain amount, such as a material/liquid ratio of more than 1:10, the collection rate of the powder continuously increased, and the bacteria survival rate rapidly dropped. Lower ratios reduced solids content, leading to incomplete droplet production and excessive moisture. Higher ratios increased viscosity, which resulted in uneven particle size distribution. Thus, 1:10 is the ideal material/liquid ratio.

#### 2.6.3. The Effect of Inlet Temperature

**Figure 10:**
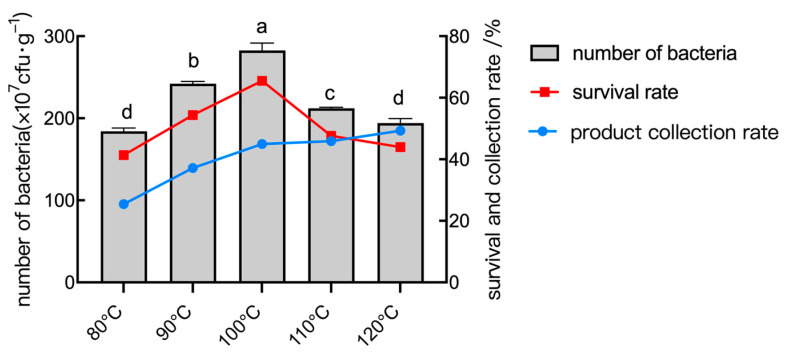
The number of bacteria, survival rate, and collection rate of the powder under different inlet temperature conditions. Data are presented as means of three replicates ± SD, and error bars represent SD for three replicates. Means with different letters have significant differences (*p* < 0.05; Duncan test).

During the spray drying process, the feed liquid was atomized and dried in contact with the hot air, and the viable bacterial count showed a decreasing trend as the temperature of the inlet air rose. As shown in Figure 10, the live bacterial count dramatically dropped at temperatures above 100 °C, suggesting that higher temperatures cause more harm to the bacteria. At lower temperatures, on the other hand, the rate of powder collection was low, the product drying was not complete, and the sticky wall phenomenon was severe. Thus, it is evident from the test that the optimal inlet temperature is 100 °C.

#### 2.6.4. The Effect of Inlet Flow Rate

**Figure 11:**
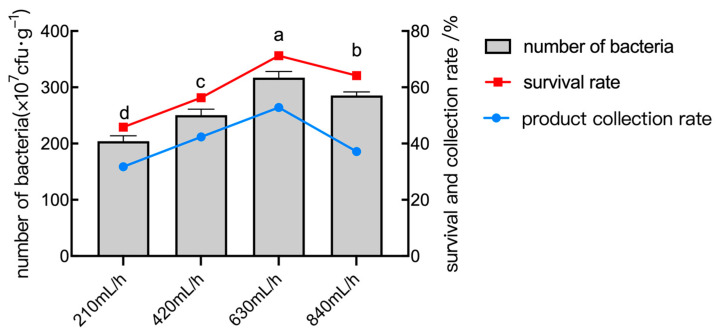
The number of bacteria, survival rate, and collection rate of the powder under different inlet flow rate conditions. Data are presented as means of three replicates ± SD, and error bars represent SD for three replicates. Means with different letters have significant differences (*p* < 0.05; Duncan test).

The inlet flow rate influenced both the spray drying efficiency and the bacterial survival rate. With an increase in inlet flow rate, the number of live bacteria and the quantity of powder collected by the product increased gradually. The inlet flow rate was too fast, which led to insufficient excessive evaporation, incomplete drying sample, and an excessively high moisture content in the product. The inlet flow rate was too slow, the atomization port was easy to be blocked, resulting in excessive evaporation, and the exit temperature was too high, which caused the faster death of the bacteria. As shown in Figure 11, the test results show that the number of live bacteria, survival rate, and powder collecting rate of the powder achieved their maximum values at an inlet flow rate of 630 mL/h.

These single-factor results guided the orthogonal design to refine the spray drying process.

### 2.7. Orthogonal Test of Spray Drying Conditions

In this test, the material/liquid ratio, inlet temperature, and inlet flow rate were selected as the optimization factors for the orthogonal test, and a three-factor three-level orthogonal test was carried out. Using the powder collection rate as the primary index, the larger the extreme difference, the greater the influence of the factor on the index, and the results demonstrate that the three factors influencing the powder collection rate were as follows: material/liquid ratio > inlet temperature > inlet flow rate. According to the results, spray drying under these conditions required the best combination of A2B2C2 (a material/liquid ratio of 1:10, an inlet temperature of 100 °C, and an inlet flow rate of 630 mL/h). The powder collection rate of *B. amyloliquefaciens* JL54 was obtained under these conditions, reaching 78.41% (See Table 5 and Table 6).

## 3. Discussion

The study of *B. amyloliquefaciens* as a biocontrol agent has received increased attention in recent years due to its broad-spectrum antifungal activity, environmental safety, and ability to induce systemic resistance in host plants [36]. There are more reports on the study of *B. amyloliquefaciens* as a biocontrol agent, which has a wide range of bacterial inhibition, and has been used extensively in the prevention and control of fungal diseases in food crops, cash crops, vegetables, and other fungal diseases, with a broad prospect for industrialization [37]. Nowadays, the majority of products on the market are aqueous agents [38], with a comparatively simple dosage form. However, the commercialization of *Bacillus*-based products still faces key challenges, particularly in achieving stable fermentation performance, high bacterial viability, and effective formulation strategies [32,33]. To develop new microbial fungicides, it is crucial to investigate the ideal spray drying process condition components and the ideal fermentation medium and circumstances for high efficiency and low cost [39]. The effective number of live bacteria is a critical factor that affects microbial agents in production. Therefore, this study aimed to enhance the bacteria activity of *B. amyloliquefaciens* JL54 and improve the applicability of the final product by systematically optimizing the fermentation conditions and spray drying process, with the goal of providing a theoretical basis and technical support for its industrial application.

In this experiment, the PB test and BBD test were applied to screen out the optimal fermentation parameters, and three major factors—yeast powder concentration, shaking time, and magnesium sulfate concentration—were identified as critical for enhancing *B. amyloliquefaciens* JL54 viability. The optimized medium and culture conditions resulted in a viable count of 4.45 × 10^9^ cfu/mL, representing a 296.67-fold increase compared with the unoptimized system. This significant improvement underscores the strain’s robust growth potential. Such a magnitude of enhancement is rarely reported in the context of *Bacillus* fermentation studies and suggests that *B. amyloliquefaciens* JL54 has considerable potential as a high-yield biocontrol strain suitable for industrial-scale production.

The experiment revealed that strains would grow differently depending on the parameters of the fermentation culture [40]. According to this experiment, yeast extract was chosen as the best carbon source, as it supports its function in increasing microbial biomass by facilitating the quick absorption of vitamins and amino acids [41]. Similar to the kind and concentration of the carbon source evaluated in this experiment, Kuang et al. [41] optimized the carbon source for *B. amyloliquefaciens* DMBA-K4’s high synthesis of extracellular polysaccharides, achieving a result of 10 g/L yeast extract. This could also because it is a component of the composite carbon source, which also contains nitrogen and other growth elements needed for bacterial growth [42]. Similarly, yeast powder outperformed other nitrogen sources, most likely due to its high peptide content, which promotes lipopeptide synthesis—a critical antibacterial metabolite in *Bacillus* spp. [20]. Additionally, yeast extract was more readily and quickly utilized by microorganisms in the fermentation medium, and, when combined with the best nitrogen source, it can further support *B. amyloliquefaciens* JL54 growth [43]. Magnesium sulfate was the most effective inorganic salt, contributing to the integrity of cellular membrane integrity and enzymatic activity [44]. Consistent with the findings of this investigation, Xie et al. [45] optimized the medium components of *B. amyloliquefaciens* L-4-3 and found that the best nitrogen source and inorganic salt were, respectively, yeast powder and magnesium sulfate. This result demonstrated that magnesium ions are essential for various metabolic functions in Bacillus spp. The beneficial effect of magnesium sulfate observed here highlights its role as a critical mineral nutrient in microbial fermentation systems.

Apart from the medium’s components, the conditions and process of fermentation also have a significant impact on the quantity of viable bacteria [46]. pH affects intracellular enzyme activity and biofilm stability and alters nutrient uptake by microorganisms [47]. The ideal initial pH for strain JL54 in this experiment was determined to be 6.4, which is comparable to the ideal pH value of endophytic *B. amyloliquefaciens* Xe01 that Zhou optimized [44]. Furthermore, the amount of inoculum has an impact on the growth and metabolic profile of microorganisms; too much inoculation will use up the medium’s nutrients and cause nutrient deficiencies later on, while too little inoculation will extend the exponential period of bacterial growth and cause fermentation to be delayed [48]. A 1% inoculum can provide sufficient biomass without depleting nutrients. The results of fermentation will also be impacted by the shaking temperature, rotational speed, and shaking time. A suitable temperature will allow microorganisms to develop quickly, while an unsuitable temperature will prevent or even kill bacteria. The bacterium itself also depends on time and temperature for energy metabolism and enzyme synthesis [49], while a shaking time of 31.8 h ensures prolonged exponential growth. These parameters were optimized further using response surface methodology (RSM), which revealed synergistic interactions between yeast powder, magnesium sulfate, and shaking time that contributed significantly to the maximized viable count. The increase in biomass achieved reflects the high adaptability and responsiveness of *B. amyloliquefaciens* JL54 to environmental conditions.

The 54% disease control efficacy observed in field experiments highlights JL54’s practical relevance. This performance parallels *Bacillus velezensis* wr8’s efficacy against citrus rot [22] but exceeds *Bacillus subtilis* T429’s control rates in similar settings [33]. Similarly, Xiong et al. reported that *B. amyloliquefaciens* JK6 achieved a biocontrol efficacy of 58.6% against tomato bacterial wilt under greenhouse conditions [50], which parallels the 54% control rate observed in our field study. The treatment groups, which received a 20-fold dilution of the bacterial suspension (4 × 10^9^ cfu/mL), exhibited a significantly lower increase in disease index compared with the control groups treated with sterile water. Specifically, while the control group’s disease index rose from 63.03 to 68.98 (Δ = 5.95), the treatment group showed only a modest increase from 36.17 to 38.87 (Δ = 2.7). This difference in disease progression suggests *B. amyloliquefaciens* JL54 not only inhibits pathogen proliferation but also mitigates disease progression through induced systemic resistance (ISR)—a trait documented in other *Bacillus* spp. [51]. Wu et al. demonstrated that *B. amyloliquefaciens* SQR9 triggered ISR in host plants through synergistic interactions between lipopeptides and polyketide compounds [52]. Furthermore, the fact that *B. amyloliquefaciens* JL54 was originally isolated from larch branches implies strong host compatibility, which may have further enhanced colonization efficiency and persistence *in planta*.

Spray drying, as a downstream processing step, was systematically optimized to preserve bacterial viability while enhancing powder recovery. According to the results of orthogonal spray drying tests, the material/liquid ratio was the primary factor influencing the powder collection rate, emphasizing the need for viscosity control to ensure uniform droplet formation—a challenge also noted in *B. subtilis* formulations [53]. The inlet temperature had a smaller effect, possibly because *B. amyloliquefaciens* is more resilient to adversity and high temperatures [54]. The optimal inlet temperature of 100 °C balances bacterial viability and drying efficiency, contrasting with lower temperatures (80–90 °C) required for less thermotolerant strains [32]. Compared with previous studies that required higher inlet temperatures (e.g., 120–130 °C) and mixed protectants (e.g., skim milk and maltodextrin) for similar viability levels [32], our process achieved 78.2% survival and 78.41% recovery at only 100 °C using a single protectant. This highlights a more energy-efficient and simplified formulation. Skimmed milk powder emerged as the most effective protective agent, consistent with its ability to form a thermoprotective matrix during spray drying [55]. However, the viable bacterial number decreased as the amount of skimmed milk powder added increased. This could be because the product’s weight increased with the addition of milk powder, which also reduced the proportion of bacteria per unit weight of product, which lowered the number of live bacteria and the survival rate [33]. Furthermore, the product recovery process was too small because the test instrument was a small laboratory spray dryer with only one product cyclone collector, compared with two or three in the industrial production recovery processes. This resulted in a low amount of viable bacteria, and some of the bacteria could not be collected by exhausting the tail gas [30].

Our study bridges fermentation optimization and formulation science, addressing gaps in *B. amyloliquefaciens* commercialization. Despite the results, certain challenges remain for industrial-scale implementation. Although earlier research concentrated on aqueous formulations [38], we demonstrate that spray-dried powders maintain biocontrol efficacy while overcoming storage and transportation limitations. However, cost-effective scaling remains a challenge, as protective agents like skimmed milk powder may elevate production expenses and limit the economic viability of large-scale production—a trade-off also observed in probiotic encapsulation. To improve economic viability, future research should investigate multi-stage drying processes (e.g., spray drying followed by vacuum or freeze drying) and explore low-cost stabilizers, such as agricultural byproducts.

## 4. Materials and Methods

**Figure 12:**
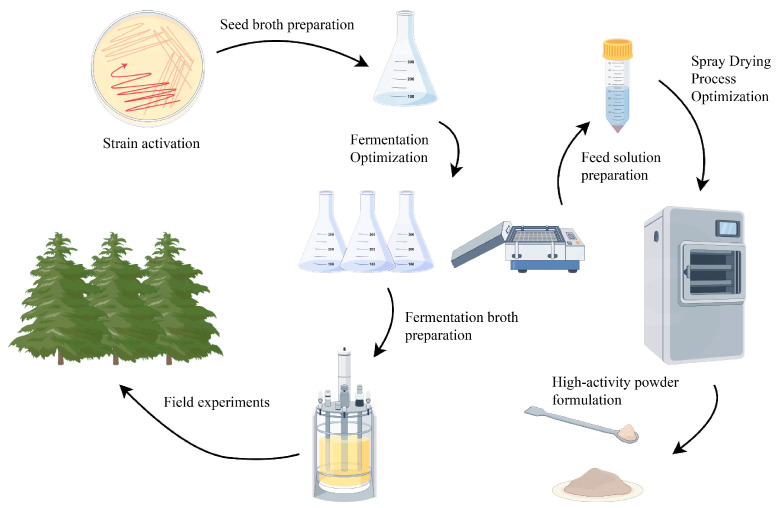
Schematic diagram of the experimental procedure.

This section details the materials used and the systematic procedures followed to optimize fermentation and spray drying processes for *B. amyloliquefaciens* JL54, and to evaluate its field efficacy. The overall experimental workflow is schematically represented in Figure 12.

### 4.1. Medium and Source of Strains

Four types of media were used in this study. NA medium: 5 g beef paste, 10 g peptone, 5 g NaCl, 20 g agar, distilled water 1 L, pH 7.2. LB liquid medium: 10 g tryptone, 5 g yeast extract, 10 g NaCl, distilled water 1 L, pH 7.4. LB solid medium: 10 g tryptone, 5 g yeast extract, 10 g NaCl, 15 to 20 g agar, distilled water 1 L, pH 7.4. Potato dextrose agar (PDA) medium: 200 g peeled potato, 20 g glucose, 20 g agar, distilled water 1 L. (Beef paste: Aobox, Beijing, China; Peptone and Agar: Solarbio, Beijing, China; Tryptone and Yeast extract: Oxoid, Hampshire, UK; Glucose and NaCl: SCR, Shanghai, China).

Each medium served a distinct function during the study. The activation of strain was achieved using the NA medium. Bacterial seed solution and one-factor basal medium were prepared using the LB liquid medium. The colony counting medium was the LB solid medium. The growth of *Neofusicoccum laricinum* (Sawada) Y. Hattori & C. Nakash was conducted using the PDA medium.

The biocontrol strain *B. amyloliquefaciens* JL54 was isolated from branches and leaves of healthy larches collected from 13 sampling sites in 8 provinces of China [34], and deposited in the Chinese Culture Collection Centre, CCTCC NO: M 2023793 (Wuhan, China). The pathogenic fungus was *Neofusicoccum laricinum* (Sawada) Y. Hattori & C. Nakash, with strain number DHKS 6-3, which is currently kept in the Forest Protection Laboratory of Nanjing Forestry University.

### 4.2. Strain Activation, Preparation of Seed Broth, and Obtaining of Fermentation Broth and Bacterial Suspension

Strain activation: to obtain a fresh and active inoculum, *B. amyloliquefaciens* JL54 was inoculated on an NA medium by the streak plate method [56] and incubated at 28 °C for 24 h to form single colonies.

Preparation of seed broth: a single colony was transferred into 100 mL LB medium with 40% liquid loading, and incubated at 28 °C and 200 rpm on a constant temperature shaker for 24 h to obtain seed broth.

Preparation of fermentation broth: The seed broth of JL54 was transferred into 100 mL conical flasks with 40% liquid volume at 1% inoculum and incubated at 28 °C and 200 rpm in a constant temperature shaker for 48 h to obtain the fermentation broth. Based on this, two experimental designs were applied to improve biomass and viable bacteria counts: single-factor tests to evaluate the impact of individual factors (e.g., carbon/nitrogen sources, pH, temperature) on bacterial growth or target product yield; response surface methodology (RSM) to model and optimize interactions between critical parameters.

Preparation of bacterial suspension: The fermented *B. amyloliquefaciens* JL54 broth was centrifuged at 8000 rpm for 10 min at 4 °C, and then poured off the supernatant. The bacterial sludge was washed twice with sterile distilled water and resuspended in a small volume of sterile water. The suspension was stored at 4 °C for later use (that same day).

Preparation of feed solution: to assess the efficacy of protectants on bacterial viability during storage or formulation, various test protectants were prepared at defined concentrations, sterilized for 20 min at 121 °C, and then thoroughly mixed with the bacterial suspension to form the spray feed solution.

Viable bacteria enumeration: Bacterial viability was quantified using the dilution spread plate method [57]. Serial dilutions were plated on LB solid medium. Plates were incubated at 28 °C for 24 h, and colonies were counted. Each sample was plated in triplicate to ensure accuracy and repeatability.

### 4.3. Optimization of Fermentation Medium and Conditions

#### 4.3.1. Optimization of Fermentation Medium

To determine the most suitable carbon, nitrogen, and inorganic salt sources for the growth of *B. amyloliquefaciens* JL54, single-factor tests were performed. This step aimed to identify medium components that significantly enhance bacterial viability.

According to the research methodology of Hong Peng et al. [33], a number of carbon sources were chosen, including maltose, mannitol, glucose, sucrose, soluble starch, and yeast extract; nitrogen sources included peptone, yeast powder, urea, soybean peptone, ammonium sulfate, and corn syrup dry powder; and inorganic salts included magnesium sulfate, sodium chloride, calcium carbonate, potassium chloride, potassium hydrogen phosphate, and sodium hydrogen phosphate. The corresponding components in the LB medium were substituted with an equal amount of the corresponding components in the base medium. *B. amyloliquefaciens* JL54 was cultivated shakingly in 50 mL conical flasks at 40% filling volume, 1% inoculum volume, 28 °C, and 200 rpm for 48 h to produce the fermentation broth. The most effective forms of carbon, nitrogen, and inorganic salts were found by calculating the quantity of live bacteria per milliliter using coating and dilution. To ascertain the ideal concentration of each component later on, the impact of each component was screened at various concentrations on the growth of *B. amyloliquefaciens* JL54.

#### 4.3.2. Optimization of Fermentation Conditions

To further improve fermentation efficiency, five critical culture parameters were individually optimized under the previously determined optimal medium composition.

The starting culture conditions were as follows: 1% inoculum, initial pH 7.0, 40% liquid loading, 28 °C shaker temperature, and 200 rpm shaker speed using the optimal medium components. To ascertain the viable bacterial population of *B. amyloliquefaciens* JL54, the five factors—inoculum amount, initial pH, liquid loading, shaker temperature, and rotational speed—were successively submitted to one-way experiments with varying setting values.

### 4.4. Response Surface Methodology (RSM) Optimization

#### 4.4.1. Plackett–Burman (PB) Test

The goal of this step was to identify the most influential factors among eight medium and process variables affecting bacterial growth. The Plackett–Burman (PB) test is a near-saturated horizontal test design method. One approach to designing near-saturated horizontal tests is the Plackett–Burman (PB) test. Its foundation is the idea of imperfectly balanced blocks, which may estimate a factor’s primary effects with the least amount of trials. This allows for the efficient and fast separation of the most significant factors for additional study from a huge number of factors that have been explored [58]. In accordance with Ma et al.’s study methodology [59], a PB test plan was created using Design Expert 10 software based on the findings of the one-factor optimization test. Three replications were set up for each set of tests, and each variable had one low level and one high level. The response value was determined by measuring the live bacteria in the JL54 strain fermentation broth. Subsequently, variables that had a notable impact on the quantity of live bacteria were eliminated.

#### 4.4.2. Steepest Climb Test

The most important influencing components can approach the maximum response area through the sharpest hill-climbing test, allowing for the determination of the ideal level combination center and additional experimental scheme optimization that will speed up the Box–Behnken design’s ability to identify the optimal response value.

The PB test findings should be used to swiftly approach the ideal area in order to obtain the center of the Box–Behnken, filter out the more significant influence variables on the number of JL54 viable bacteria, and define the climbing direction and step length. The optimal area is quickly approached to obtain the center of the Box–Behnken design (BBD).

#### 4.4.3. Box–Behnken Design (BBD)

The BBD test was carried out based on the variables and concentrations screened by the PB test and the steepest climb test. Design Expert 10 software was used to assess and interpret the test data. Each variable was tested at three levels around the central point determined from the steepest ascent. A total of 17 runs were performed, and the viable bacterial count served as the response. The data were analyzed using ANOVA, and a second-order polynomial regression model was constructed to predict the response.

### 4.5. Determination of the Growth Curve of B. amyloliquefaciens JL54

To evaluate the effect of fermentation optimization on bacterial growth dynamics, growth curves of *B. amyloliquefaciens* JL54 were generated under pre- and post-optimization conditions.

To obtain the seed solution, the conserved *B. amyloliquefaciens* JL54 was activated and inoculated in the LB liquid medium, and then incubated at 37 °C and 200 rpm for 24 h. Strain JL54 seed liquid was cultivated for 72 h at 37 °C and 200 rpm after being inoculated at 1% of the medium before and after optimization. Samples were collected every 2 h, and the *B. amyloliquefaciens* JL54 growth was monitored by measuring optical density at 600 nm (OD600) using a UV-Vis spectrophotometer [60].

### 4.6. Data Analysis and Processing

IBM SPSS Statistics 26.0 was used to perform analysis of variance and Duncan’s multiple comparisons (*p* < 0.05); response surface modeling, regression analysis, and contour plot generation were performed using Design Expert 10.0; Graphpad Prism 9.0 was used for graphing.

### 4.7. Field Experiments

A two-year (2023–2024) preventative test was carried out in the forest of Heishi Township, Dunhua City, Yanbian Korean Autonomous Prefecture, Jilin Province, during the commencement of larch dieback, to assess the efficacy of *B. amyloliquefaciens* JL54 in controlling larch dieback disease under natural conditions.

The field survey method’s sampling strategy was randomized. Two sample plots with identical cultivation conditions were chosen to create two treatment groups; three standard sample plots were chosen at random within each treatment group’s boundaries; and thirty larch trees were chosen at random from each standard sample plot. The treatments for the field efficacy test were as follows. The test sample consisted of a 20-fold dilution of the *B. amyloliquefaciens* JL54 bacterial culture at a concentration of 4 billion cfu/mL, with water serving as the blank control. Before the onset time, the test was drone-sprayed with the bacterial solution, and, 60 days following the spraying, the disease index was examined. Larch dieback grading criteria were as follows: level 0: no disease on the entire plant; level 1: less than 5% incidence of needle leaves; level 2: 6% to 25% incidence of needle leaves; level 3: 26% to 50% incidence of needle leaves; level 4: 51% to 75% incidence of needle leaves; and level 5: more than 76% incidence of needle leaves.

Disease index and control effect formulations are as follows:

Disease index = [∑ (representative value of disease level × number of diseased plants at each level)]/(representative value of the highest level × total number of plants surveyed) × 100;

Control effect = [(disease index of negative control group − disease index of treatment group)/disease index of negative control group] × 100%.

### 4.8. Single-Factor Test of the Main Parameters of Spray Drying

In the spray drying test, the factors that influenced the product collection rate and the quantity of viable bacteria were the type of protectant (Factor A), material/liquid ratio (Factor B), inlet temperature (Factor C), and inlet flow rate (Factor D). The factors are shown in Table 7.

Six protectants were tested: skimmed milk powder, β-cyclodextrin, soluble starch, cornstarch, glucose, and sucrose. Each was dissolved in water, sterilized at 121 °C for 20 min, and mixed with bacterial suspension prior to drying.

The feed broth was spray-dried using a laboratory spray dryer, with each parameter set individually while other conditions were held constant. The resulting powder was collected and evaluated for viable bacterial count (cfu/mL), survival rate (%), and powder collection rate (%).

### 4.9. Orthogonal Optimization Test

To identify the optimal combination of spray drying parameters, a 3-factor, 3-level orthogonal experiment was conducted using factors such as material/liquid ratio, inlet temperature, and inlet flow rate. Each trial was evaluated for powder recovery rate. The factor levels are shown in Table 8.

## 5. Conclusions

This study establishes a comprehensive framework for optimizing the fermentation and spray drying processes of *B. amyloliquefaciens* JL54, positioning it as a sustainable biocontrol agent against larch dieback disease.

Through Plackett–Burman testing and response surface methodology design, the optimal medium composition (12 g/L yeast extract, 11.8 g/L yeast powder, 7.2 g/L MgSO_4_) and culture conditions (30% loading, 1% inoculum, 37 °C, pH 6.4, 31.8 h shaking) achieved a viable count of 4.45 × 10^9^ cfu/mL—a 296.67-fold increase over unoptimized systems. Screening of media components and conditions demonstrated significant strain-specific variations in optimal culture parameters, necessitating tailored optimization for individual strains [61]. This highlights the strain’s robust metabolic adaptability and the efficacy of statistical design in process optimization.

Field experiments demonstrated a 54% reduction in disease progression, validating *B. amyloliquefaciens* JL54’s potential for practical application. This performance aligns with biocontrol benchmarks for *Bacillus* spp. while addressing the urgent need for eco-friendly larch disease management.

The orthogonal-optimized spray drying parameters (1:10 material/liquid ratio, 100 °C inlet temperature, 630 mL/h feed flow) achieved a 78.41% powder recovery rate, surpassing previous reports for similar formulations [30]. Skimmed milk powder proved critical for bacterial thermotolerance, underscoring its utility in industrial-scale production.

Biocontrol fungicides are important for controlling pathogenic bacteria, activating the plant immune system, and improving crop quality [39]. The application of biocontrol fungicides is growing due to the growing demand for sustainable forestry and environmentally friendly pharmaceuticals [53]. While this study establishes a robust framework for scaling *B. amyloliquefaciens* JL54 as a biocontrol agent, several obstacles need to be overcome in order to connect laboratory productivity with ecological and industrial sustainability. High-quality protective agents (e.g., skimmed milk) may elevate production costs. Industrial adoption requires cost-effective stabilizers without compromising bacterial viability [62]. The reliance on high-cost protective agents like skimmed milk powder highlights the need to explore low-cost alternatives, such as agricultural byproducts, to enhance economic viability without compromising bacterial viability. Furthermore, the long-term stability of the spray-dried formulation under variable environmental conditions—such as humidity, temperature changing, and UV exposure—remains untested, necessitating accelerated aging studies and adjuvant integration to enhance field resilience, while also requiring further investigation into appropriate dosage forms for forest conditions. Scaling laboratory-optimized parameters to industrial bioreactors and spray dryers requires pilot-scale validation [63], especially to address oxygen transfer constraints and thermal stress in large-scale systems. 

In addition to improving JL54’s commercial viability, addressing these gaps will contribute to global efforts in sustainable forestry, which will lessen dependency on chemical fungicides while preserving ecological integrity. Further research should combine biological control and interdisciplinary cooperation to transform *B. amyloliquefaciens* JL54 from a promising candidate into a practical scalable strategy for larch shoot blight management.

## Figures and Tables

**Table 1:** Analysis of variance for each factor in the PB trial. * indicates that the model is statistically significant at *p* < 0.05.

Source of Variance	Coefficient Estimate	Stdized Effec	Sum of Squares	Degrees of Freedom	Mean Square	F	*p*	Contribution/%	Inference	Importance Ranking
Model			10,964.00	8	1370.50	25.69	0.0110		*	
A: yeast extract concentration	8.28	16.57	823.20	1	823.20	15.43	0.0294	7.37		5
B: yeast powder concentration	16.36	32.73	3212.78	1	3212.78	60.21	0.0045	28.77		1
C: magnesium sulfate concentration	−13.45	−26.89	2169.49	1	2169.49	40.66	0.0078	19.43		3
D: initial pH	4.59	9.18	252.73	1	252.73	4.74	0.1178	2.26		7
E: inoculum volume	4.15	8.29	206.26	1	206.26	3.87	0.1440	1.85		8
F: shaking bottle time	15.28	30.56	2802.66	1	2802.66	52.53	0.0054	25.10		2
G: shaking bottle temperature	−7.07	−14.15	600.24	1	600.24	11.25	0.0439	5.38		6
H: loading volume	8.64	17.29	896.66	1	896.66	16.80	0.0263	8.03		4

**Table 2:** Steepest climb test.

Treatment No.	Yeast Powder Concentration/g/L	Shaking Time/h	Magnesium Sulfate Concentration/g/L	Number of Bacteria/ (×10^9^ cfu·mL^−1^)
1	8	24	3	2.22
2	9	26	4	3.02
3	10	28	5	3.16
4	11	30	6	3.5
5	12	32	7	4.05
6	13	34	8	3.27

**Table 3:** BBD ANOVA results.

Source	Sum of Squares	Degrees of Freedom	Mean Square	F	*p*	Inference
Model	9.48	9	1.05	11.69	0.0019	**
A: yeast powder concentration	0.45	1	0.45	5.01	0.0602	ns
B: shaking time	0.02	1	0.02	0.22	0.6518	ns
C: magnesium sulfate concentration	1.05	1	1.05	11.67	0.0112	*
AB	0.09	1	0.09	1.00	0.3508	ns
AC	0.063	1	0.063	0.69	0.4323	ns
BC	0.01	1	0.01	0.11	0.7487	ns
A^2^	2.08	1	2.08	23.07	0.002	**
B^2^	0.77	1	0.77	8.54	0.0222	*
C^2^	4.23	1	4.23	46.98	0.0002	**
Residual	0.63	7	0.09			
Lack of fit	0.082	3	0.027	0.2	0.891	ns
Pure error	0.55	4	0.14			
Cor total	10.11	16				

Note: ** means strongly significant (*p* < 0.01); * means significant (*p* < 0.05); ns means not significant.

**Table 4:** Field trial of *B. amyloliquefaciens* JL54 for the control of larch dieback disease.

Treatments	Standard Sample Plot	Sample Number	2023 Disease Index	2024 Disease Index
Treatment	1	1–30	36.60	44.00
	2	31–60	36.60	41.30
	3	61–90	35.30	31.30
Control	1	91–120	58.0	64.67
	2	121–150	64.60	70.67
	3	151–180	66.50	71.61

Note: in this table, treatments 1, 2, and 3 are 20-fold dilution of the JL54 bacterial culture at a concentration of 4 billion cfu/mL; controls 1, 2, and 3 are clear water as the blank control.

**Table 5:** Spray drying orthogonal test results.

Trial No.	A: Material/Liquid Ratio	B: Inlet Temperature	C: Inlet Flow Rate	Powder Collection Rate/%
1	1	1	1	54.96
2	1	2	3	56.18
3	1	3	2	51.8
4	2	1	3	77.84
5	2	2	2	78.41
6	2	3	1	69.5
7	3	1	2	39.24
8	3	2	1	35.19
9	3	3	3	27.65
K1	162.94	172.04	159.65	
K2	225.75	169.78	169.45	
K3	102.08	148.95	161.67	
R	41.22	7.70	3.27	

**Table 6:** Spray drying orthogonal test ANOVA results.

Source	Sum of Squares	df	Mean Square	F	*p*	Inference
A	2549.26	2	1274.63	771.61	0.001	**
B	108.02	2	54.00	32.69	0.030	*
C	17.85	2	8.925	5.40	0.156	ns
Error	3.304	2	1.652			

Note: ** means strongly significant (*p* < 0.01); * means significant (*p* < 0.05); ns means not significant.

**Table 7:** Single-factor level used in this study.

Factors	Factor A	Factor B	Factor C	Factor D
1	Skimmed milk powder	1:20	80 °C	210 mL/h
2	β-Cyclodextrin	1:10	90 °C	420 mL/h
3	Soluble starch	1:5	100 °C	630 mL/h
4	Cornstarch	3:10	110 °C	840 mL/h
5	Glucose		120 °C	
6	Sucrose			

**Table 8:** Spray drying orthogonal test factor levels in this study.

Level	Factors		
	Material/Liquid Ratio	Inlet Temperature	Inlet Flow Rate
1	1:20	95	420
2	1:10	100	630
3	1:5	105	840

## Data Availability

The original contributions are included in the article. Further queries can be directed to the corresponding author.

## References

[1] Hattori Y., Ando Y., Nakashima C. (2021). Taxonomical re-examination of the genus *Neofusicoccum* in Japan. Mycoscience.

[2] Kazuo I. (1964). A New Lecture on the Illustration of Forest Trees.

[3] Zhao J.Z., Yu W.X., Wang N.Y. (1995). Current status of domestic and international research on larch shoot blight. For. Sci. Technol..

[4] Sawada K. (1950). Fungi of Conifers in Tohoku district (2)-fungi of Conifers other than cedar. J. For. Sci. Technol..

[5] Yamamoto W., Kazuo I. (1961). Species of the genera of Glomerella and Guignardia with special reference to their imperfect stages. Sci. Rep. Hyogo Univ. Agric..

[6] Sun M.L., Huang L., Ye J.R., He J., Wang Z. (2022). Advances on pathogenic mechanisms and endophytes-employed biological control of fungal diseases on major timber forests in China. J. Nanjing For. Univ. (Nat. Sci. Ed.).

[7] CSFA (2013). State Forestry Administration Announcement (no. 4, 2013) National Forestry Quarantine Pest List. https://www.gov.cn/gzdt/2013-01/15/content_2312345.htm.

[8] SFGA (2023). State Forestry and Grassland Administration Notice (No. 567), ‘List of Priority Invasive Alien Species for Management’. http://www.moa.gov.cn/govpublic/KJJYS/202211/t20221109_6415160.htm.

[9] EPPO (2022). Neofusicoccum laricinum (GUIGLA) Associated EPPO Standards.

[10] Xu Z.G. (2009). General Plant Pathology.

[11] Liu M.G., Zhang D., Lu W.Y., Wang J. (2018). Pathogen of Pine Shoot Blight: Research Progress. Pathog. Pine Shoot Blight Res. Prog..

[12] Jorge P., Andrea R.R., Rosa M.M., Alejandra F.M. (2021). Microorganisms as biocontrol agents against bacterial citrus diseases. Biol. Control.

[13] Yavuz E., Gunes H., Harsa S., Bulut C., Yenidunya A.F. (2004). Optimization of pulsed field gel electrophoresis (PFGE) conditions for thermophilic bacilli. World J. Microbiol. Biotechnol..

[14] Ling P.S., Pirasannah E., AlAdil B.M.M., Mohd Y.N., Ahmad K.W.N.I.W., Mohamad A.M.S., Aqlima A.S., Nurbaya O.S., Sooa L., Suriana S. (2023). Antimicrobial peptides from *Bacillus* spp. and strategies to enhance their yield. Appl. Microbiol. Biotechnol..

[15] Dai L., Li L., Liu Y., Shi Y., Cai Z. (2021). Whole genome sequencing and genomics analysis of *Bacillus amyloliquefaciens* BS-3 with biocontrol activity. Microbiol. China.

[16] Qin X., Xiao Y., Xiong Q., Kong W.L., Borriss R., Gao Z., Fan B. (2025). Four antimicrobial compounds and ISR induction are involved in biocontrol of crown gall disease by the plant beneficial rhizobacterium *Bacillus velezensis* FZB42. Plant Dis..

[17] Ying T.T., Wu P.J., Gao L.L., Wang C.C., Zhang T.H., Liu S.S., Huang R.Q. (2022). Isolation and characterization of a new strain of *Bacillus amyloliquefaciens* and its effect on strawberry preservation. LWT.

[18] Michael B., Danai E., Sabrina C., Peter B., Roger S., Monika E.S., Sophia J. (2022). Whole Genome Sequencing Reveals Biopesticidal Origin of *Bacillus thuringiensis* in Foods&#13. Front. Microbiol..

[19] Pan X.H., Chen F., Guo X.P., Wang Y.L., Cui Z.Q., Huang T.P., Hou Y.M., Guan X. (2024). Development of a safe and effective *Bacillus thuringiensis*-based nanobiopesticide for controlling tea pests. J. Agric. Food Chem..

[20] Kamal M.M., Savocchia S., Lindbeck K.D., Ash G.J. (2016). Biology and biocontrol of *Sclerotinia sclerotiorum* (Lib.) de Bary in oilseed Brassicas. Australas. Plant Pathol..

[21] Femina C.C., Senthil K.P., Tsopbou N.P. (2021). A review on new aspects of lipopeptide biosurfactant: Types, production, properties and its application in the bioremediation process. J. Hazard. Mater..

[22] Wang R., Lan B.F., Zhou L.Q., Yang H., Meng J.Z., Chen W.C. (2023). Optimization of fermentation conditions of *Penicillium* sp. antagonistic bacterium *Bacillus velezensis* wr8 in postharvest citrus by response surface method. China Brew..

[23] Zhang C.F., Si H.L., Chen L., Liang X.J., Zhang J.L. (2020). Isolation and identification of nicosulfuron-degrading enzymes from *Bacillus velezensis* CF57. J. Hebei Agric. Univ..

[24] Chen Z., Huang J., Zhao J., Hao Y.S., Liang H. (2018). Isolation and Identification of Pathogenic Fungi of Strawberry Root Rot and the Inhibition of Antagonistic Bacteria CM3 on These Fungi. Biotechnol. Bull..

[25] Chai Q.K., Zhang B., Chang R.K., Liu H.Q., Tian X.W., Wang Y.H. (2019). Preliminary study on the effect of the induced resistance in cucumber with *Bacillus amyloliquefaciens* LJ02 against *Botrytis cinerea*. Acta Phytopathol. Sin..

[26] Hou B.H., Xu B.L., Xun T., Zhang S.W. (2017). Determination of the stability and antibacterial spectrum of antibacterial substances of the *Bacillus amyloliquefaciens* TS-1203 against *Valsa mali*. J. Gansu Agric. Univ..

[27] Wang J., Wang Z., Liu C., Song M., Xu Q., Liu Y., Yan H. (2024). Genome analysis of a newly isolated *Bacillus velezensis*-YW01 for biodegrading acetaldehyde. Biodegradation.

[28] Wang S.W., Wang Q.H., Zheng C.X., Liu X., Li B. (2023). Advance in the drug form, preparation and applicationof *Bacillus amyloliquefaciens*. J. Sci. Teach. Coll. Univ..

[29] Jin W.J., Zhang F.J., Zhang X.M., Yu J.J., Zou L., Sun B.S., Yan Y.Z., Xue J. (2021). Bacteriostatic and antiseptic effects of *Bacillus amyloliquefaciens* B15 wettable powder. Jiangsu Agric. Sci..

[30] Qian Y.X., Kang J.C., Luo Y.K., Geng K., Lei B.X., Zhang B. (2016). Development of Wettable Powder of *Bacillus amyloliquefaciens* X17 for Control of *Botrytis cinerea* on Kiwifruits. Chin. J. Biol. Control.

[31] Cai X.C., Liu J.D., Gao X., Wang B.T., Liu C.H. (2015). Preparation of Endophyte *Bacillus amyloliquefaciens* CC09 WP. Mod. Agrochem..

[32] Huang M., Yao L., Zhao L.H. (2015). Research on Microencapsulation of Linseed Oil by Spray Drying. Farm Prod. Process..

[33] Meng X.K., Yu J.J., Yin X.L., Nie Y.F., Yu M.N., Chen Z.Y., Liu Y.F. (2014). Study on the Spray Drying Technology of Antagonistic Bacteria *Bacillus subtilis* T429. Chin. J. Biol. Control.

[34] Liu Y.Q., Han S.J., Song L.W., Li L.M., Wang H.F., Pan M., Tan J.J. (2024). Screening of bacterial endophytes of larch against *Neofusicoccum laricinum* and validation of their safety. Microbiol. Spectr..

[35] Pan M., Wang Y., Tan J., Liu F., Hu J. (2023). Optimization of Fermentation Conditions for *Bacillus pumilus* LYMC-3 to Antagonize *Sphaeropsis sapinea*. Fermentation.

[36] Wei H., Liu S.H., Song Q.F., Bao F., Shang Q.X., Liu Z.P., Wei Y.M. (2009). Studies on Fermentation Process of Strain BJ-6 of *Bacillus amyloliquefaciens*. Chin. Agric. Sci. Bull..

[37] Li Y., Gao Z., Kong W., Xiao Y., Adjei M.O., Fan B. (2025). Biocontrol of Crown Gall Disease of Cherry Trees by *Bacillus velezensis*. Plants.

[38] Yang Z.S., Guo N.Q. (2001). Research on spray drying process of pesticide water dispersible granules. Pesticides.

[39] Peng X.Q., Wang R., Xu X.L., Fan H.M. (2014). DVS meitauza starter preparation by spray drying process. China Brew..

[40] Li P.T., Hsiao W.L., Yu R.C., Chou C.C. (2013). Effect of heat shock on the fatty acid and protein profiles of *Cronobacter sakazakii* BCRC 13988 as well as its growth and survival in the presence of various carbon, nitrogen sources and disinfectants. Food Microbiol..

[41] Kuang J.H., Huang Y.Y., Hu J.S., Yu J.J., Zhou Q.Y., Zhao S., Liu D.M. (2020). Optimization of fermentation conditions and anti-oxidation of exopolysaccharide produced by *Bacillus amyloliquefaciens* DMBA-K4. Food Ferment. Ind..

[42] Xia B.H., Zhao J., Gong P., Han X.B., Peng Y.L., Liu K., Wang C.Q., Ding Y.Q., Du B.H. (2021). Optimal Fermentation Conditions for *Bacillus amyloliquefaciens* DSYZ Based on Response Surface Methodology. J. Shandong Agric. Univ. (Nat. Sci. Ed.).

[43] Song K.D., Li Z.J., Han K.C., Cui M.Y., Zhou M.M. (2019). Characteristics of traditional dough fermentation starter (Jiaozi) made from different size corn flour. Food Sci. Technol..

[44] Zhou X.P., Shu C.H., Teng K., Gan Z.D., Xiao Q.M., Chen w., Li X.H., Liu T.B. (2020). Identification and Fermentation Optimization of Antagonistic *Bacillus amyloliquefaciens* Xe01. Chin. Tob. Sci..

[45] Xie L.J., Xiao L., Wu S.D., Cheng W. (2022). Optimization of Fermentation Conditions for *Endophytic Bacillus amyloliquefaciens* L-4-3. Hunan Agric. Sci..

[46] Zhang W., Wei L., Xu R., Lin G., Xin H., Lv Z., Qian H., Shi H. (2020). Evaluation of the Antibacterial Material Production in the Fermentation of *Bacillus amyloliquefaciens*-9 from Whitespotted Bamboo Shark (*Chiloscyllium plagiosum*). Mar. Drugs.

[47] Wiggins E.H., Mann P.F.E., Trevelyan W.E., Harrison J.S. (1952). Effect of pH on the synthesis of cell carbohydrate during fermentation by Baker’s yeast. Biochim. Biophys. Acta.

[48] Gibbons W.R., Westby C.A. (1986). Effects of inoculum size on solid-phase fermentation of fodder beets for fuel ethanol production. Appl. Envion. Microbiol..

[49] Yang Q.H. (2020). Optimization of culture medium and fermentation conditions of *Bacillus amyloliquefaciens* CQN-2 strain from fishery sources. J. Fish. Res..

[50] Xiong H., Li Y., Cai Y., Cao Y., Wang Y. (2015). Isolation of *Bacillus amyloliquefaciens* JK6 and identification of its lipopeptides surfactin for suppressing tomato bacterial wilt. RSC Adv..

[51] Wei D.P., Ye J.R., Liang M.J. (2020). Spray drying processes of *Bacillus valeriana* YH-18. J. Nanjing For. Univ. (Nat. Sci. Ed.).

[52] Wu G., Liu Y., Xu Y., Zhang G., Shen Q., Zhang R. (2018). Exploring Elicitors of the Beneficial Rhizobacterium *Bacillus amyloliquefaciens* SQR9 to Induce Plant Systemic Resistance and Their Interactions with Plant Signaling Pathways. Mol. Plant-Microbe Interact..

[53] Ma X., Huang Y., Cheng J., Wang W. (2015). Microencapsulation of *Bacillus subtilis* and its control of *Rhizoctonia solani* damping-off of tomato. Chin. J. Pestic. Sci..

[54] Ma D. (2018). Optimization of Solid-state Fermentation Process and Development of Biocontrol Agent from *Streptomyces microflavus*. ResearchGate.

[55] Wu D.L., Wang Z.G., Mei L., Xue X.H. (2015). Optimization of process parameters for the preparation of microcapsules of lactic acid bacteria by vacuum low-temperature spray drying method. Jiangsu Agric. Sci..

[56] Liu F., Li Y.Y., Chen W., Cao C.X., Wen S.H., Rao B., Huang D.Y. (2023). Optimization of Fermentation Medium for Spore Production by *Bacillus velezensis* YC11 through Response-Surface Methodology. Chin. J. Biol. Control.

[57] Gong J.H., Wang J. (2018). Introduction to the method of enumerating viable bacteria by dilution-coated plate method. Teach. Biol..

[58] Hu S., Mei L.H., Yao S.J. (2003). Optimization of nattokinase liquid fermentation by response surface methodology. Food Ferment. Ind..

[59] Ma H.Z., Yan M., Li L., Yang Z.P., Zhang J.X., Meng Q.X., Shi X.Y. (2023). Optimization of Fermentation Parameters of *Bacillus velezensis* C44 Based on Response Surface Methodology. J. Shanxi Agric. Sci..

[60] Liu J., Zhang C.Z., Zhao H. (2023). Optimization of medium and fermentation conditions of *Bacillus velezensis* P9 resistant to *Fusarium oxysporum*. China Brew..

[61] Shi H.M., Ye J.R., Wang M., Lu L.X., Shi J.W. (2023). Optimizing spore-producing medium and culture conditions of *Bacillus velezensis* strain YH-18 by response surface methodology. J. Nanjing For. Univ. (Nat. Sci. Ed.).

[62] Wang X., Gao J., Gao Y., Zhang L., Xu C., Li Q., Li L., Xue J. (2024). Analysis of surfactant production by *Bacillus cereus* GX7 and optimization of fermentation conditions. Colloids Surf. B Biointerfaces.

[63] Xiao H.Q., Li Y.Z., Zhao M.M., Lin Q.L., Liu J., Zhou Q., Jiang M.J. (2020). Optimization of Spray Drying Parameters for the Probiotic Powder Preparation of *Bacillus subtilis* Prob 1822. Sci. Technol. Food Ind..

